# A Brief Review

**Published:** 1893-01

**Authors:** Benjamin Lord

**Affiliations:** New York


### A BRIEF REVIEW.[1]

BY DR. BENJAMIN LORD, NEW YORK.

It will be remembered that Dr. I. B. Davenport, of Paris, some five years ago contributed to our Society what was regarded as an able paper on the teeth and jaws, in which he considered quite fully the question of the retention or extraction of the sixth-year or first permanent molars.

At the annual meeting of the British Dental Association, held at Manchester, England, in August last, the principal subject considered, as I understand it, was the treatment of the particular teeth just alluded to, and at that time Dr. Davenport delivered an

[1] Read before the New York Odontological Society, November 15, 1892.

address in which he expressed views practically identical with those contained in his paper read before this Society,—viz., advising the retention of those teeth in almost every case, and giving his reasons in full for such an opinion.

I do not propose, by any means, to go over any considerable part of the discussion of this subject on the occasion named; but I have thought that some allusions to it would be of more or less interest, and perhaps profit.

The discussion, which was entered into by a goodly number of the members of the Association, took a wide range, as was natural in view of the preliminary remarks of the president, Mr. H. C. Quinby, who, by the way, is an American, and I believe very well known to many in this Society.

I may quote a few sentences from the remarks of Mr. Quinby at the opening of the discussion. He says, "This subject may be described, generally, as the treatment of irregularities and overcrowding of the human teeth. But the special subject for our discussion this morning (one which is necessarily well represented in this museum) is the tendency of overcrowding to promote the destructive effects of caries; and whether it may be justifiable as a conservative measure, in cases where we see indications of danger to the youthful dentine from this cause, to remove an upper and a lower tooth from each side of the mouth, with a view to the giving of more space to the anterior teeth, permitting them to separate, and, as nearly as possible, isolate themselves, thereby preventing the inception of carious disease in such teeth as have not already been attacked. If such a measure is justifiable, which teeth should be selected for extraction, so as to give the necessary relief, and when would be the best time for the extracting to secure the best results for the future of the mouth?"

Dr. Davenport, in his further study of the subject, said at the beginning of his address, "The discussion of the question of extraction of the sixth-year molars I have always approached from a general stand-point, leaving out my wearisome search amidst the darkness and chaos of imperfect organs and imperfect results of individual cases, where, if one were found which seemed to be explained or explicable, or referable to a principle, the next would very likely contradict it."

I may here say that I believe that all who will take the trouble to read Dr. Davenport's address will find in it much that is valuable.

The discussion of the subject by the various members of the

Association who took part in it showed a great deal of interest and much well-considered thought. It also exhibited a degree of intelligence that could only come from a careful study of the question under consideration; and we are forced to concede that they are better students over there than we are here.

An important aid in the discussion, and particularly in illustrating the various effects of treatment, was furnished by a large number of carefully-made models, contributed by many dentists and arranged by Mr. G. G. Campion. It was decided, however, that in order best to show and illustrate the effects of extraction, whether favorable or not, casts should be made, both before and after the operation.

It must occur to us all, Mr. President, that there is here a suggestion and a lesson that we should do well to take up and act upon, in order to derive the greatest advantage, both for ourselves and for those who shall come after us. Can we not at once set about securing models of all cases where extraction is resorted to or found to be imperative, both before and after the operation?

In this communication scarcely any idea can be given of the scope of this discussion; but I am very glad to be able to say that the strongest arguments were in favor of retaining all the teeth that can possibly be preserved. Of course, however, when we come to the treatment of abnormal conditions, we are often obliged to choose between the least of two evils.

I believe that the consensus of opinion was that in cases where it is found imperative to remove the sixth-year molars, it should by no means be done until after the full eruption and occlusion of the twelfth-year molars; this is certainly, in my opinion, a most wise practice, although some would extract much earlier. All seem to have been in favor of extracting antagonizing teeth, and to have held that, whenever it is decided, for any cause, to remove a molar from either the upper or the lower arch, the opposing or occluding tooth should also be removed. I believe, on the other hand, that it is better to lose one tooth than two, and that, as a rule, there will be less loss of grinding surface from the tilting or tipping of the teeth, if the opposing teeth are allowed to remain. There was not a word said about preserving the roots of the sixth-year molars in cases where the crowns could not be saved. I think it preferable, in all cases where the roots can be preserved in a healthy, or even a fairly healthy condition, to do so; as the tipping of the remaining teeth is thereby prevented, and it is the tipping of the teeth which causes more loss of masticating surface than results from the loss of the crown that has broken away. Other evils also follow the removal of the roots, such as the shortening of the arch, and the weakening of the adjoining teeth from the loss of the alveoli.

Mr. Quinby, the newly-elected president of the Association, closed as well as opened the discussion. For the most part he is much in favor of extracting as a conservative treatment; and he endeavors to justify his opinion by giving a great many reasons for it, and by presenting the subject in many aspects. I may read two or three sentences from his remarks, giving his views and practice in regard to extracting, which I believe, however, are not altogether justified by the experience of those of us who have given the most thought and attention to the subject.

He says in his address, in regard to his official position and his recommendation in practice, "I stand here, to-day, in the most responsible position in which you can place me, and because of this,—because what I say here will have more emphasis than it could have from any other place in the gift of the profession,—because I have been asked by students whether, after all the adverse arguments by some of my American colleagues, I still adhere to my doctrine of prevention by space,—I want to repeat from this chair the advice I have so often given to young practitioners when they meet with cases of rapid approximal decay in the permanent incisors and bicuspids, at or before the twelfth year, and especially when the family history indicates delicate teeth. It is this: Do not have the slightest hesitation in recommending the extraction of the sixth-year molars as soon as the twelfth-year molars are sufficiently advanced to be in occlusion, the object being to give space for distinct separations between all the anterior teeth."

Mr. Quinby's inaugural has many good points, and contains much that we might do well carefully to consider, and grow wiser and better by the consideration. He is severe in some of his criticisms and denunciations of the conduct and practice of some among us, and of the so-called "American" dentist abroad; but perhaps he has seen and known enough to warrant all that he says. Yet it may be that he, being an American, has felt at liberty to say more than he otherwise would, more perhaps than some of his English confrères would endorse.

It is not to be presumed that America wishes to be, or should be, held responsible for all dentists who call themselves "American" dentists. Some of our schools have no doubt graduated men who know next to nothing about dentistry, practically; and if they have thus disgraced themselves and the profession, the responsibility and the odium should be placed where they belong. I will quote a few sentences further from Mr. Quinby, which are near the commencement of his address:

"I trust you will pardon me if I call your attention for a few minutes to some of the causes of the change that has taken place in the estimation in which American practice is held now, compared to what it was forty years ago. I wish to speak of some of the phases of what I must call unprofessional conduct, which have brought discredit upon American dentists abroad and at home,—a discredit which is deeply felt by all the earnest, conscientious members of our profession (and it is scarcely necessary to say that there are many such in America), who will always jealously defend the interests and prestige of our specialty, both there and everywhere."

After discussing and denouncing the practice of securing patents for various kinds of implements, methods, and appliances, he says, "These are, I believe, some of the reasons why the word 'American,' used as a prefix to dentistry, constitutes almost a term of reproach, for on this side of the Atlantic it has become, I am sorry to say, synonymous with the veriest chicanery and humbug; but America has not ceased, and I hope will never cease, to produce dentists who are honorable men, and who will cordially agree with the sentiments of a late letter in the *Times* by a distinguished member of this Association, who says, 'Dentistry, like medicine and surgery, is catholic, and is practised by honest men for the public good, and therefore all its methods are made public to all members of the profession.'"

Dr. Quinby's address contains almost a complete record of the many objectionable features connected with the practice of dentistry all over the world by the "camp-followers" and imitators of American dentists. Bogus diplomas, patented instruments and methods, the sale of State and county patent-rights, advertisements of peculiar treatment and claims of special skill in the public press, all receive merited and scathing denunciation at Mr. Quinby's hand.

Remembering the official action of the New York Odontological Society against the patenting of instruments or methods, and because of the exclusion of all reference to the same from our published proceedings, I feel that I but express the sentiments of at least a large majority when I commend Mr. Quinby for all that he has said against such unprofessional conduct.

I also take pleasure in assuring him that the true American dentist,—by which I mean the skilful, conscientious, tooth-saving practitioner,—joins him in condemning all such practices, and would inform him that by both example and precept, and by stringent dental law, the effort is being made, and with fairly satisfactory results, to rid the profession in this country of these objectionable features.

It is strange that so good a thinker as Mr. Quinby evidently is should have overlooked the great forces which are at work for the purification of the dental profession in the United States, among which the Dental Protective Association and the excellent dental laws of many of our States naturally come first; and the labors of Dr. H. C. Meriam in his crusade against the patent evil must not be forgotten.

Unfortunately, I think, for Mr. Quinby's logic, he has found in his well-arranged list of professional sins committed by quacks and charlatans, masquerading as American dentists, an excuse for a rather wholesale condemnation of American dentistry at large. But we find that in practical life the most successful men have the greatest number of imitators; the best artist's pictures are copied; the most pleasing architectural results are followed in the reproduction of cheaper and less meritorious works; and all because of that never-failing homage which men of all countries and professions, with native shrewdness, pay to success and skill.

Were the standard of practical dentistry as high in any other country as it is in America, quacks and non-professionals would now be masquerading as Russian dentists or Swiss dentists, or, begging Mr. Quinby's pardon, as English dentists.

May it not be considered surprising that Mr. Quinby, being an American, should give expression to such views, notwithstanding that England has been his adopted country for many years, and that the influence of environment can be understood? Yet, is he justified, even at his distance from his native land, in expressing his opinions of the status of the profession here in such sensational phrases?

I have already in this little paper paid a merited tribute to the scholarly attainments of English dentists, and I am quite ready to agree with Mr. Quinby's high estimate of their qualifications, so far as theoretical knowledge is concerned; but it is to be hoped that his words of praise will not cause them to slacken their efforts to add to their manipulative skill.

